# Interaction of the Gulf Stream with small scale topography: a focus on lee waves

**DOI:** 10.1038/s41598-020-59297-5

**Published:** 2020-02-11

**Authors:** Charly de Marez, Noé J. Lahaye, Jonathan Gula

**Affiliations:** Université de Bretagne Occidentale, CNRS, IRD, Ifremer, Laboratoire d’Océanographie Physique et Spatiale (LOPS), IUEM, Brest, France

**Keywords:** Fluid dynamics, Physical oceanography

## Abstract

The generation of lee waves in the Gulf Stream along the U.S. seaboard is investigated using high resolution realistic simulations. The model reproduces the surface signature of the waves, which compares favourably with observations from satellite sun glitter images in the region. In particular, a large number of internal waves are observed above the Charleston Bump. These waves match well with the linear theory describing topographically-generated internal waves, which can be used to estimate the associated vertical transport of momentum and energy extracted from the mean flow. Finally, small scale topographic features are shown to have a significant impact on the mean flow in this region of the Gulf Stream, and the specific role of lee waves in this context is outlined.

## Introduction

Large-scale currents impinging on the topography can generate numerous physical processes, including internal waves –so-called lee waves–, which extract energy from the current and radiate it away from the bottom. For instance, in the Drake Passage, the energy transport due to a lee wave has been estimated locally at a peak rate of *O*(1) W m^−2^ ^1^. The impact in the global ocean is significant as lee waves are thought to extract about 20% of the global wind power input to the geostrophic circulation^2^. Half of this energy is dissipated in the water column, within a kilometer of the topography^2^, when waves break and induce turbulent mixing^3^. Hence, lee waves provide a significant mechanism for the transfer of energy from large-scale flows to turbulent length scales^4^. Lee waves also redistribute momentum from the mean flow and can affect the large-scale circulation through the generation of a drag^5,6^. Numerical models have thus to take into account the redistribution of momentum and energy, by using parameterizations of the wave drag and energy dissipation^7^.

**Figure 1:**
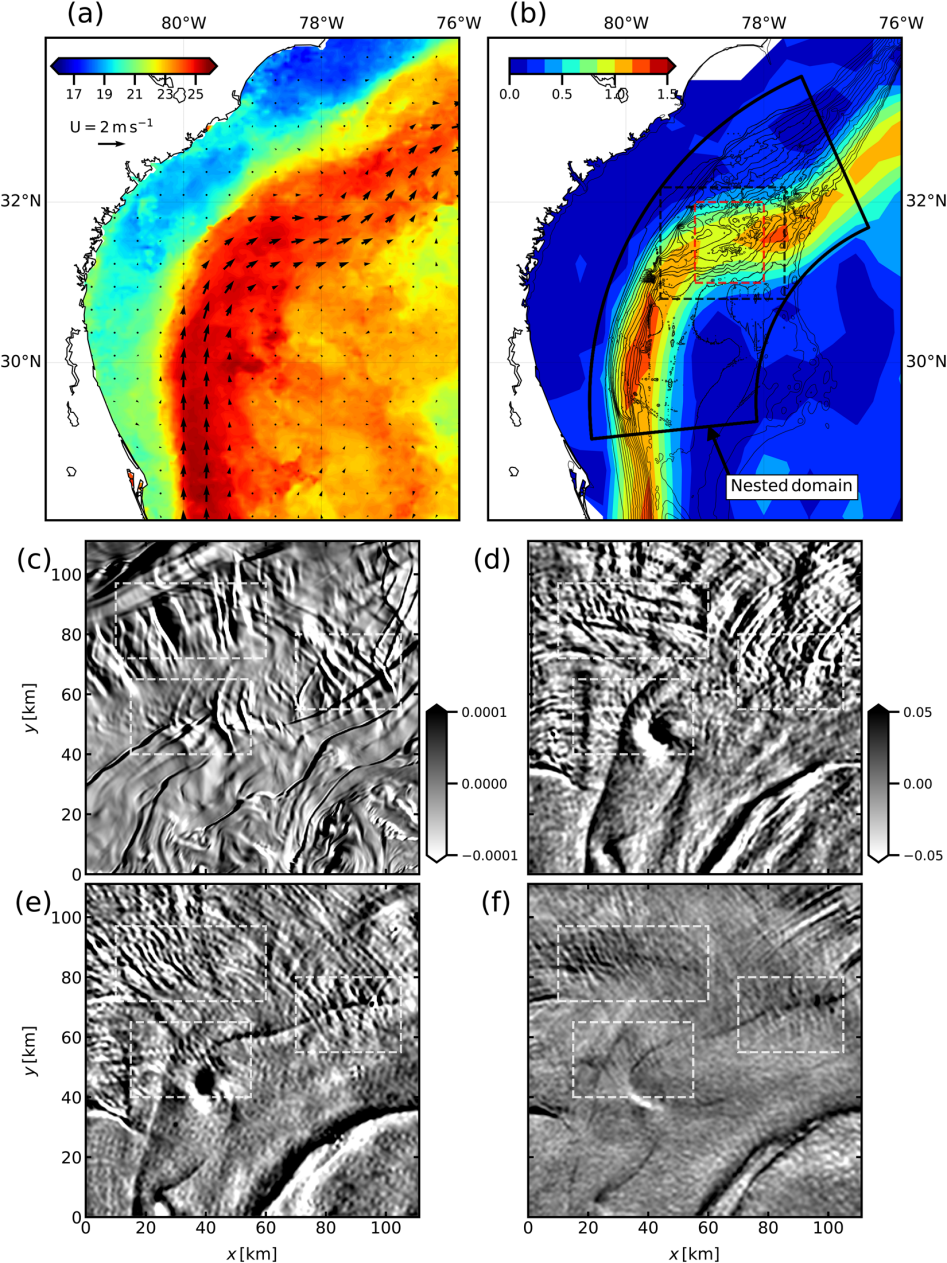
(**a**) Sea Surface Temperature [in °C] over the Gulf Stream region on 2010/04/01^27^ and surface currents from AVISO on 2010/03/31. (**b**) Geostrophic surface currents norm from AVISO on 2010/03/31 [in m s^−1^], black contours are isobaths from the surface to 1000 m depth every 50 m. The red dashed rectangle indicates the area of the zoom presented in panels (**c**–**f**); the black dashed rectangle indicates the area where we then show the lee waves related quantities. (**c**) Estimation of the surface roughness [in s^−1^] in the LEEWA simulation. The coordinate (0, 0) corresponds to 79°W 31°N. (**d**–**f**) Surface roughness [in arbitrary units] from Sun-glitter measured on 2010/04/01 by 3 different satellites (TERRA, AQUA, and Envisat) with (MODIS, MODIS, and MERIS) instruments in the same area than (**c**). White dashed rectangles indicate places where qualitatively similar roughness patterns appear in both the simulation and the satellite observations. The rightmost rectangle corresponds to the position of Hoyt Hills, where the same kind of wavefront-like pattern has already been observed^12^.

The Gulf Stream flows northward along the U.S. continental shelf from the Strait of Florida to Cape Hatteras, see Fig. 1(a,b). Its path is mainly controlled by the bottom topography and boundary shape^8^. The current is strong, with a maximum at the surface of about 2.5 m s^−1^ ^9^. Near 31.5° N, 79° W, a prominent topographic feature is present off the coasts of South Carolina and Georgia: the Charleston Bump. Above it, the flow is shallow (*O*(500) m) and intense (*O*(1) m s^−1^) even at the bottom, thus providing a context of strong current-topography interaction^10^ and conditions that are favorable for the generation of lee waves^11^.

Internal waves can be observed using synthetic aperture radars (SAR) or sun glitter images through their surface roughness signature. Over the Charleston Bump, quasi-stationary wavefront-like patterns in the surface roughness have been attributed to lee waves generated through the interaction of the Gulf Stream with seamounts^12^. Similar signals have also been observed in this area through their impact on patchiness in seabird distribution^13^. More recently, measurements using sea-gliders have confirmed the presence of lee waves along the path of the Gulf Stream near the Charleston Bump^11^.

In this paper, we investigate the dynamics of lee waves and, more generally, the role of small-scale topography on the mean current, with a focus on the Charleston Bump region. Our results rely on regional realistic simulations of the Gulf Stream using very high horizontal and vertical resolution (see section 2.2) that allows us to resolve small-scale topographic features (*O*(1) km) as well as vertical motions in the range 10–100 m. We address the following questions: (1) Are lee waves generated in the high resolution simulations of the Gulf Stream? (2) What are their structure and their properties? (3) How the small scale topographic features impact the mean flow of the Gulf Stream? In a first part we present some elements of theory considered for the study of lee waves and the numerical setup of the simulation we used. Then we analyze the lee wave generation in the path of the Gulf Stream using the simulation and theory. Finally we discuss the impact of the small topographic features on the mean current.

## Methods

### Linear theory of lee waves

The equation for the propagation of lee waves can be obtained by linearizing the incompressible primitive equations around a steady state. Following ref. ^5^, we first neglect the background rotation to keep the equations simple. This assumption is reasonable for monochromatic topography of horizontal lengthscale *O*(<10) km (a criterion which is satisfied where we compare the solution with the model outputs). Seeking steady solutions (${\partial }_{t}\to 0$), the vertical displacement of a streamline $\eta$ is defined such that $w=U{\partial }_{x}\eta$, where *w* is the vertical velocity within the fluid and *U*(*z*) is the mean horizontal current, oriented along the *x*-axis. The lee wave problem in the horizontal Fourier space (*k*, *m*) thus reads:

1 $${\partial }_{zz}\,\tilde{\eta }(k,m,z)+{n}^{2}\,\tilde{\eta }(k,m,z)=0,$$

supplemented by a radiation condition at the top of the domain and a Dirichlet condition at the bottom:

2 $$\tilde{\eta }(k,m,z=0)=\tilde{h}(k,m).$$

Here, $\tilde{h}$ (resp. $\tilde{\eta }$) is the Fourier transform of the topography *h* (resp. the streamline $\eta$), and $n(k,m,z)$ can be interpreted as a local vertical wavenumber of the solution.

In the case of lee waves, the condition for a hydrostatic flow is:

3 $$\varepsilon =\frac{2\pi U}{NL}\ll 1\to {\rm{Hydrostatic}}$$

where *L* is the typical length of the topographic features (equal to the horizontal wavelength of the waves). Under this regime, the dispersion relation for a hydrostatic lee wave is^5^:

4 $${n}^{2}(k,m,z)=\frac{{k}^{2}+{m}^{2}}{{k}^{2}}(\frac{{N}^{2}}{{U}^{2}}+\frac{{\partial }_{zz}U}{U}),$$

where $N(z)$ is the Brunt-Väisälä frequency. In specific regions of the ocean such as the Charleston Bump area, the vertical shear of the mean current is large, with variations of current up to 2 m s^−1^ over 500 m depth. In these conditions, $\frac{{\partial }_{zz}U}{U}\sim \frac{{N}^{2}}{{U}^{2}}$, and the shear of the mean flow must be taken into account in the dispersion relation. In the following, the 3D theoretical prediction of hydrostatic lee waves is obtained by numerically solving Eq. (1) using the WKBJ approximation^14^, with the bottom condition (2) and the dispersion relation (4).

Finally, we briefly recall the criteria for the linear propagation of lee waves in the ocean. The rotation of the Earth imposes a lower bound to the range of horizontal wavenumbers *k* for which waves can propagate. In terms of the non-dimensional number $\varepsilon$, the radiation condition in a rotating and stratified fluid is^2^:

5 $${\rm{\Pr }} < \varepsilon $$

where Pr = *f*/*N* is the Prandtl number and *f* is the Coriolis frequency. Without the hydrostatic approximation, an additional upper bound $\varepsilon  < 1$ is present.

The importance of nonlinear effects associated with lee waves can be estimated using a “lee wave Froude number”^15^ (or “steepness parameter”^16^). It is given by:

6 $${{\rm{Fr}}}_{{\rm{lee}}}=\frac{NH}{U}=\frac{{\rm{vertical}}\,{\rm{velocity}}\,{\rm{within}}\,{\rm{the}}\,{\rm{lee}}\,{\rm{wave}}}{{\rm{group}}\,{\rm{velocity}}\,{\rm{of}}\,{\rm{the}}\,{\rm{lee}}\,{\rm{wave}}},$$

where *H* is the scale of the bathymetry height. It follows that:

7 $$\begin{array}{lll}{{\rm{Fr}}}_{{\rm{lee}}} &  <  & 1\to {\rm{linear}}\,{\rm{propagation}}\,{\rm{of}}\,{\rm{the}}\,{\rm{waves}}\\ {{\rm{Fr}}}_{{\rm{lee}}} & \gtrsim  & 1\to {\rm{non}}-{\rm{linearity}}\,{\rm{of}}\,{\rm{the}}\,{\rm{waves}}.\end{array}$$

### Model setup

**Figure 2:**
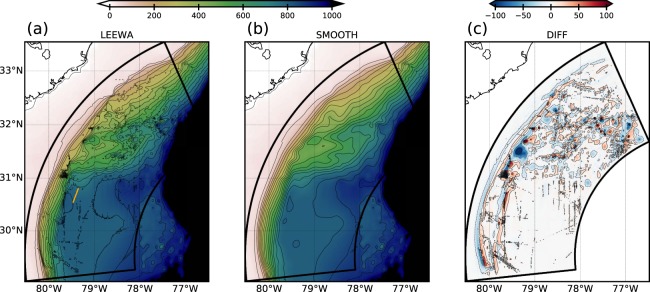
Bathymetry [in m] from the SRTM30_PLUS dataset used in (**a**) the LEEWA simulation and (**b**) the SMOOTH simulation; the orange straight line in (**a**) indicates the position where we then compare the model with the linear theory. The difference [in m] between the two bathymetries is displayed in (**c**).

To study the generation of lee waves, we performed realistic simulations in the Gulf Stream region using the Coastal and Regional Ocean COmmunity model (CROCO^17^). It solves the hydrostatic primitive equations for the velocity **u**, the temperature *T* and the salinity *S*, using a full equation of state for seawater^18^. A one-way nesting approach is used, with successive horizontal grid resolutions of Δ*x* ~ 750 m for the parent, and $300\,{\rm{m}} > \Delta {\rm{x}} > 150\,{\rm{m}}$ for the child simulations. The parent simulation has been shown to accurately represent the Gulf Stream Dynamics and the hydrography along the U.S. seaboard^8^. In this paper we study outputs of the child simulations. The nested domain, shown in Fig. 1(b), consists of a grid of 2050 × 1026 points. The time step is chosen so that it respects the Courant–Friedrichs–Lewy condition, $dt=30\,{\rm{s}}$. Sub-grid parameterizations are identical than in the parent simulation; see section 2 in ref. ^8^ for further details. In particular, vertical mixing of tracers and momentum is done using a K-profile parametrization (KPP), and the effect of bottom friction is parameterized through a logarithmic law of the wall. Simulations have 128 terrain-following vertical levels, which are stretched such that the resolution increases near the surface and the bottom; for instance, over the Charleston Bump, Δ*z* ~ 2 m at the surface, Δ*z* ~ 8 m at 500 m and Δ*z* ~ 6 m at the bottom. The simulations are initialized by interpolating a snapshot of the parent simulation on the child grid (on 2016/03/20). They are forced at the lateral boundaries by the parent simulation, and at the surface by a monthly varying wind, which limits the generation of near-inertial waves. There is no tidal forcing (and hence no internal tides). Two simulations have been run, hereafter named LEEWA and SMOOTH, with two different bathymetries. The latter are constructed from the SRTM30_PLUS dataset and are smoothed using a Gaussian kernel, with a width of 4 grid points for LEEWA and 50 grid points for SMOOTH. This avoids aliasing, and retains small scale topographic features only in the LEEWA simulation. The bathymetries used for the two simulations are shown in Fig. 2. For both simulations, we considered a 4-days spin-up period. All diagnostics shown, including time-averages over the whole simulation, are performed after this spin-up, during a one-month long period (the time of the simulations), which is long enough for studying lee waves because they adjust rapidly to the flow.

## Lee Waves in the Gulf Stream

In this section, we show that our numerical simulations produces lee waves and describe their dynamics. We first provide a qualitative comparison of their surface signature against SAR images, then we present more quantitative results on the lee wave dynamics and their impact on the flow based on diagnostics of the simulations and linear theory.

### Surface signature

Streak-like patterns of the surface roughness have been observed by synthetic aperture radars (SAR) in the path of the Gulf Stream and interpreted as the surface signature of lee waves by ref. ^12^. Similar surface roughness patterns can be seen in more recent sun glitter images collected on 2010/04/01 by three different satellites over the Gulf Stream region, as shown in Fig. 1(d–f) and Fig. 1(a) of Supporting Information. The data have a resolution of 250 m and are band-passed between 2 and 25 km. They show roughness streak-like patterns upstream and over the Charleston Bump, where the intense current flows over rough topography (see Fig. 1(a,b)). Patterns typical of lee waves and similar to the ones described in ref. ^12^ are visible over Hoyt Hills, see Fig. 1(d–f). The signal also includes the signature of submesoscale fronts and filaments advected by the Gulf Stream, which can be identified in the SST field. An unstable filament breaking up into submesoscale cyclonic vortices (described in ref. ^19^) is visible between the bottom left and the right side of the satellite images.

Here, we compare the observed surface roughness patterns to the outputs of a simulation, showing that the latter qualitatively reproduces the signature of lee waves observed at the surface by sun glitter images. To this aim, we compute the quantity $({\partial }_{x}+{\partial }_{y})\,(u+v)$ at the surface in the LEEWA simulation (Fig. 1(c) and Fig. 1(b) of Supporting Information). Indeed, the surface roughness observed in sun glitter images can be interpreted as^20^:

$${\rm{roughness}}\sim \alpha {\partial }_{x}u+\beta {\partial }_{y}u+\gamma {\partial }_{x}v+\delta {\partial }_{y}v,$$

where the coefficients *α*, *β*, *γ* and *δ* are functions of the wind direction and the position of the measuring device with respect to the position of the sun. Setting the coefficients to 1 gives a first approximation of the surface roughness in the simulation. As in the satellite observations, fronts related to mesoscale and submesoscale turbulence within the simulation are visible throughout the domain.

**Figure 3:**
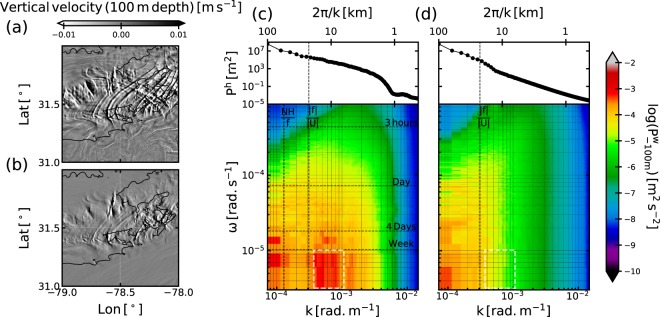
(**a**) (resp. (**b**)): Snapshot of vertical velocity (resp. time-lowpass-filtered vertical velocity) at 100 m depth in the LEEWA simulation on 2016/04/10 (after 22 days of simulation). Black contours are the isobath at 600 m. (**c**) (resp (**d**): $(k,\omega )$ Power Spectral Density of the vertical velocity at 100 m depth in the $(k,\omega )$ space (bottom panels) in the LEEWA (resp SMOOTH) simulation. The power spectrum of the bathymetry used in the simulation are represented in the top panels. Vertical dashed lines indicate |*NH*|/| *f*| and | *f*|/|*U*|, which are estimated using spatial averages of the stratification, height, bottom velocity and Coriolis frequency. |*NH*|/| *f*| is the first Rossby radius of deformation indicating the typical horizontal extension of mesoscale eddies. White dashed rectangles indicate the area of the spectrum where we expect to spot the signature of lee waves.

Roughness patterns can be seen upstream and over the Charleston Bump in the simulation, similarly as in the satellite observations, see Fig. 1(b) of Supporting Information. More precisely, wavefront-like patterns are visible over the Charleston Bump and over Hoyt Hills (Fig. 1(c)). More quantitative comparisons between model outputs and observations are made difficult by the fact that (1) the details (including the wavelength) of topographic features may differ between the bathymetry used in the simulation and the real one, and (2) the apparent wavelength of the wavelike patterns in the sun glitter images may be misleading due to the unknown transfer function of the measurement device – see the discrepancies between the images from three different satellites in Fig. 1. However, this comparison shows that the simulation produces wavefront-like patterns at locations that are consistent with the satellite images. We should stress that these patterns are persistent in time (the three satellite images were taken at 6 h intervals), which is consistent with topographically-generated disturbances. These wave patterns are also visible in the vertical velocity field at 100 m depth (Fig. 3(a)), *i*.*e*. below the thermocline (which is about *O*(50) m depth in the Gulf Stream). This reinforces the hypothesis that these patterns in the simulations are most likely induced by lee waves, which is further confirmed in the following.

### Properties of the lee waves

Dispersion diagrams ($(k,\omega )$ spectra) of the variance of vertical velocity at 100 m depth are calculated in both simulations above the Charleston Bump and shown in Fig. 3(c,d) (see Supporting Information for more details on the calculation). Azimuthally averaged power spectra of bathymetry for the two simulations are also shown in Fig. 3(c,d). An energetic region in the $(k,\omega )$ space is clearly visible in the LEEWA simulation spectrum (dashed white rectangle in Fig. 3(c,d)) at spatial scales of *O*(10) km and temporal scales of *O*(>1) weeks. This is a signature typical of lee waves, which are expected to be quasi-stationary and associated with spatial scales lower than | *f*|/|*U*|, as follows from the condition (5). The slow temporal variations of the lee waves are due to the oscillation of the Gulf Stream between a deflected and a weakly deflected position over the Charleston Bump^8^. Looking at the SMOOTH bathymetry spectrum, one can see that topographic features of length scale lower than | *f*|/|*U*| have been cut-off. According to the theory (see section 2.1), lee waves can not propagate under these conditions. Hence, there should not be such waves in the SMOOTH simulation, which is confirmed by the absence of the corresponding energetic patch in the $(k,\omega )$ spectrum.

**Figure 4:**
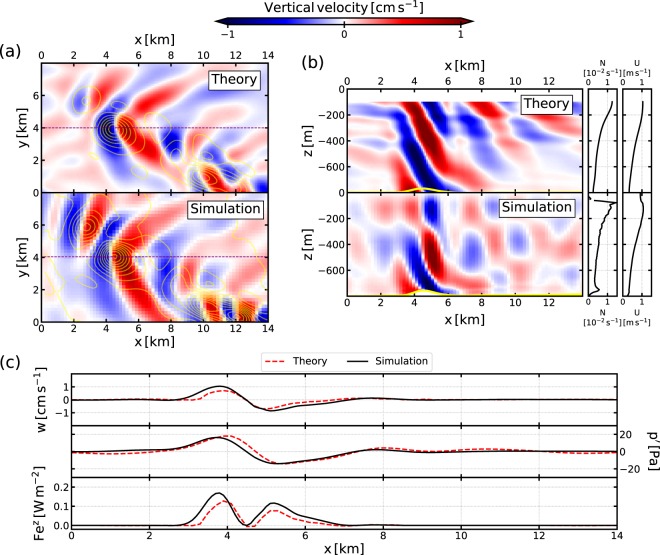
Comparison between the 3D hydrostatic linear theory and time-lowpass outputs from the LEEWA simulation (around the location presented by the orange line in Fig. 2(a)). (**a**) Horizontal sections at 500 m depth of vertical velocity. Yellow contours indicate 5 m-separated isobaths. (**b**) Vertical sections taken above an isolated seamount (see purple line in (**a**)) and profiles of stratification *N* and along-section velocity *U*. Bold yellow line indicates the bathymetry. (**c**) Values of vertical velocity *w*, pressure anomaly *p*′ and vertical energy flux *Fe*^z^ at the bottom of sections (**b**).

**Figure 5:**
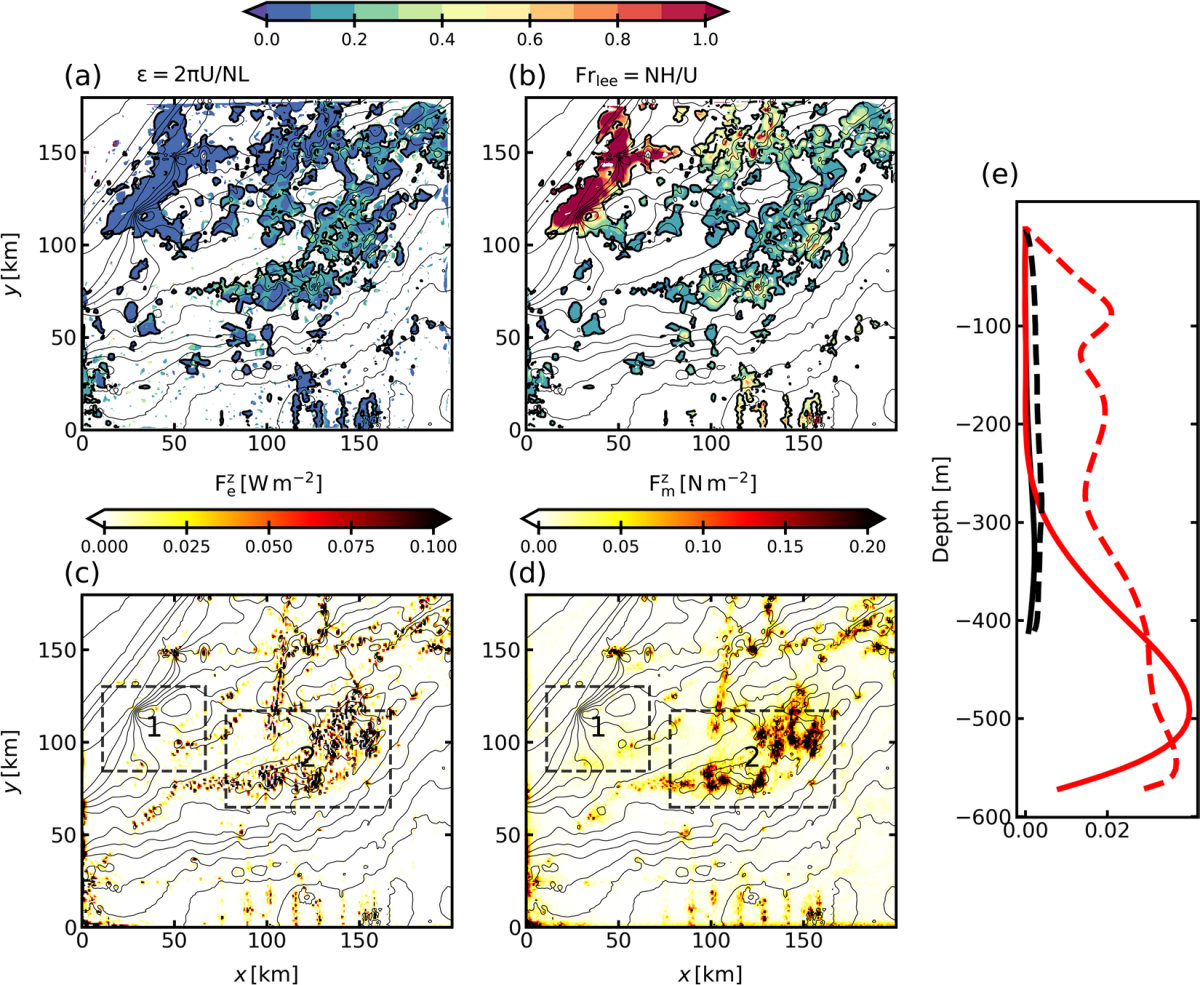
Maps of (**a**) radiation parameter $\varepsilon$, (**b**) lee wave Froude number Fr_lee_, (**c**) vertical flux of energy ${{\rm{F}}}_{{\rm{e}}}^{{\rm{z}}}$, and (**d**) vertical flux of momentum ${{\rm{F}}}_{{\rm{m}}}^{{\rm{z}}}$, associated with the generation of lee waves over the Charleston Bump in the LEEWA simulation. (**a** and **b**) are computed between the surface mixed-layer and the bottom mixed-layer, while (**c** and **d**) are mean values at the bottom (in the first 200 m); (**a**–**d**) are time-averaged over the whole simulation. The coordinate (0, 0) corresponds to 79.5°W 30.8°N. Integrating ${{\rm{F}}}_{{\rm{e}}}^{{\rm{z}}}$ and ${{\rm{F}}}_{{\rm{m}}}^{{\rm{z}}}$ over the domain shown gives respectively a net flux of energy of 0.3 GW and a net flux of momentum (equivalent to the form drag induced by lee waves) of 0.6 GN. (**e**) Timely and horizontally averaged profiles of ${{\rm{F}}}_{{\rm{e}}}^{{\rm{z}}}$ [in W m^−2^] (solid), and ${{\rm{F}}}_{{\rm{m}}}^{{\rm{z}}}$ [in N m^−2^] (dashed) in area 1 (black) and 2 (red) shown in (**c**,**e**).

The shape of the lee waves within the LEEWA simulation is compared with the 3D linear solution (see section 2.1). To do so, we first isolate lee waves in the simulation: quasi-stationary dynamical features are extracted using a fourth-order low-pass Butterworth filter. The cutting period is chosen at 4 days to retain the slow variations of the current (*O*(1) week) while removing all rapid (*O*(<1) day) motions; this period is justified by the fact that lee waves have a temporal scale lower than 4 days (see Fig. 3(c)). In particular, low-pass filtered vertical velocity, density and horizontal velocities are used to compute the vertical energy and momentum fluxes (shown in Figs. 4(c) and 5(c–e)). An example of such filtering can be seen in Fig. 3(a,b). The comparison is shown in Fig. 4 along a typical section (indicated by a bold orange line in Fig. 2(a)). The section is centered around an isolated seamount and tangential to the flow. Along the section, $U\sim {10}^{2}\times N$, the topography has a magnitude *H* of order *O*(<50) m, and a typical lengthscale of about 2 km, such that the dynamics is hydrostatic and linear, and the rotation can be neglected. The theoretical prediction is computed using smoothed profiles of *U* and *N* from the simulation (shown in Fig. 4(b)) and a de-trended and windowed topography.

The horizontal structure of the lee wave generated by the seamount at $(x,y)\sim (5,4)$ km in the model (Fig. 4(a)) is very similar to the theoretical prediction. The vertical structure also matches well with the theoretical prediction (Fig. 4(b)) in the lower half of the domain. In particular the orientation of the phase lines and the intensity of the vertical velocity at the bottom (Fig. 4(c)) are in good agreement. The shape and intensity of the pressure anomaly *p*′ calculated from the simulation outputs (see Supporting Information) are also very similar to the theoretical predictions (Fig. 4(c)), with a positive pressure anomaly upstream and a negative one downstream. The vertical energy flux $F{e}^{z}=p{\prime} w$^5^ is found positive with a peak value of $F{e}^{z}=O(0.1)\,{\rm{W}}\,{{\rm{m}}}^{-2}$ (Fig. 4(c)), which agrees with the theoretical prediction and confirms the *a priori* expectation that energy is radiated upward.

While the horizontal structure and amplitude of the waves match well with the theory near the bottom, the patterns near the surface are different. This is likely due to the reflection of the waves at the free surface and the associated modal structure of the waves, which are not taken into account in our 3D linear solution. These modes are clearly visible here because the thickness of the fluid layer is very close to 2*πU*/*N* (the theoretical vertical wavelength of hydrostatic lee waves). The fact that the bottom still presents the characteristics of upward propagating waves may be because the waves lose energy near the surface, thus reducing the amount of energy propagating downward in the deeper part of the water column. A possible cause is that wave energy is redistributed to the mean current through *e*.*g*., imbalanced processes near the surface, where the vertical shear of the current is large^21^.

The generation process and the amplitude of the lee waves within the simulation can be reasonably well described by linear hydrostatic theory along the section of Fig. 4. Comparisons with theory along other vertical sections show the same overall agreement. In particular, the depth-averaged “lee wave Froude number” (defined in Eq. (6)) is lower than unity over most of the Charleston Bump (Fig. 5(b)). Moreover, the “radiation parameter” $\varepsilon$ (defined in Eq. (3) and shown in Fig. 5(a)) satisfies both the radiation condition and the hydrostatic condition (${\rm{\Pr }}\ll \varepsilon \ll 1$) above most of the important topographic features over the Charleston Bump. Note that the smoothing of the topography (see section 2.2) implies that the typical length of the seamounts is always greater than 1 km, which is the main reason why the later inequality is mostly satisfied.

In some (limited) locations where the current is intense and the fluid is weakly stratified, we observe $\varepsilon =O(1)$, thus implying that the hydrostatic assumption does not stand there and that the numerical model reaches its limits. Also, some regions of the Charleston Bump have topographic features with a typical horizontal lengthscale too large to enable the generation of propagating lee waves, thus showing the impact of the scale of topographic features on the amount of energy radiated into lee waves. This is the case in the small area 1 shown in Fig. 5, where the fluxes of energy and momentum are close to zero, in opposition to the small area 2 where lee waves propagate, see Fig. 5(e).

Hence, we have shown above that lee waves can propagate and are hydrostatic and linear in most of the domain. The above conclusion allows us using linear hydrostatic theory to draw more quantitative conclusions on the lee waves dynamics in this region. The total energy converted into lee waves over the Charleston Bump area (computed in the LEEWA simulation) is 0.3 GW (corresponding to a mean value of ${F}_{e}^{z}=8\,{10}^{-3}\,{\rm{W}}\,{{\rm{m}}}^{-2}$ in the bottom 200 m, see Fig. 5(c)), which is again in good agreement with a linear estimate using ref. ^2^’s method, which yields a total conversion of *O*(0.3) GW (not shown). Finally, we estimate the form drag exerted by lee waves on the mean flow following^14^:

$${F}_{D}={\rho }_{0}\sqrt{{(u{\prime} w)}^{2}+{(v{\prime} w)}^{2}},$$

where *u*′ and *v*′ are estimated by keeping only the small-scale variations (similarly to the pressure anomaly *p*′, see Supporting Information). *F*_*D*_ is equivalent to the vertical flux of momentum radiated by lee waves ${{\rm{F}}}_{{\rm{m}}}^{{\rm{z}}}$, shown in Fig. 5(d). Integrated over the Charleston Bump, this gives a drag of *O*(1) GN. It represents about 10% of the form drag exerted by the whole Charleston Bump (${\rm{O}}(10)\,{\rm{GN}}$, from ref. ^8^) or 30% of the viscous drag exerted by the whole Charleston Bump (${\rm{O}}(3)\,{\rm{GN}}$, from ref. ^8^). Note that calculation of the form drag over non-isolated bathymetry is quite sensitive to the subdomain chosen for evaluating the bottom pressure anomaly. Personal calculations (not shown) show that the corresponding value could be lower than the estimate by ref. ^8^, meaning that the fraction attributed to lee waves can be taken as a lower bound. This shows anyway that lee waves have a significant impact on the Gulf Stream’s mean flow, over the Charleston Bump.

## Hydrodynamical Impact of Small Scale Topographic Features

The diagnostics discussed in the previous section show that lee waves with physically-consistent properties are generated in the LEEWA simulation. Although probably dominant in this region of strong current, lee waves are not the only physical mechanism through which small scale topographic features impact the mean current (other processes include *e*.*g*. the generation of larger scales evanescent waves and small-scale turbulence). In this section, we discuss this aspect more generally by comparing the two simulations (LEEWA and SMOOTH) with different (resp. rough and smooth) topography.

**Figure 6:**
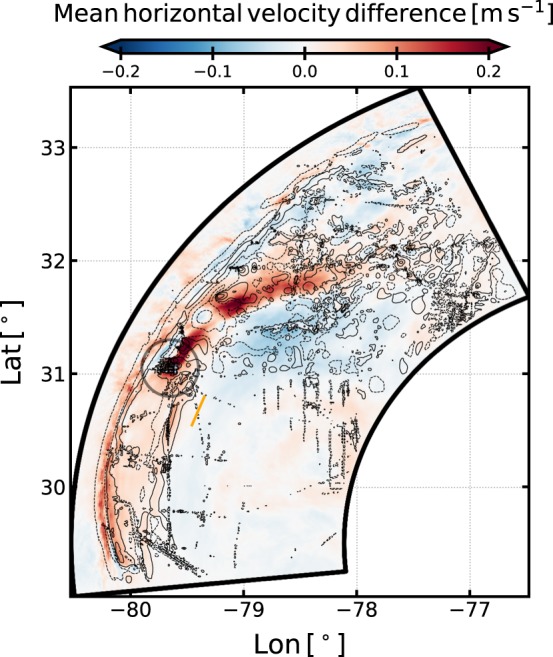
Timely and vertically averaged difference of the horizontal velocity magnitude between the SMOOTH and the LEEWA simulations (a positive pattern means that the velocity in the SMOOTH simulation is larger than in LEEWA). Black contours are differences between the bathymetry used in the two simulations, at values [±10, ±50, ±100] m, orange straight line indicates the position of the section discussed in Fig. 4.

The mean horizontal flow is significantly different between the two simulations. The timely and vertically averaged difference between horizontal velocity amplitudes in the SMOOTH and the LEEWA simulations is shown in Fig. 6. The difference reaches up to 0.2 m s^−1^ in the areas where the largest seamounts have been smoothed. The dipolar structure of the difference indicates that it corresponds to a modification of the path of the Gulf Stream. The mean current is shifted northward in the SMOOTH simulation and forms a less pronounced meander. Mean velocity profiles over the Charleston Bump show that the velocity difference reaches up to 1 m s^−1^ near the surface (not shown). This difference is most likely due to hydrodynamical control of the flow by the shape of the seafloor, since topography is the only difference between the simulations. Therefore, the mean current of the Gulf Stream, over a one-month period, is strongly impacted by small scale seamounts with horizontal size of *O*(<20) km and vertical extent of *O*(<100) m.

It would be speculative to attribute the difference in the mean flow between the two simulations to the impact of lee waves only, as other processus – *e*.*g*. evanescent lee waves^16,22^ and nonlinear motions– may play a role as well, in places such as area 1 in Fig. 5. Nonetheless, it has been shown in the previous section that lee waves exert a significant drag on the current in that region (more than 10% of the total drag). Lee waves impact the vertical structure of the flow by extracting kinetic energy at depth (cf. Fig. 5), and subsequently redistributing (and partially dissipating it) through wave-mean flow interactions^21^ or nonlinear processes (including breaking). The less pronounced meander in the SMOOTH simulation is consistent with the behavior observed in lower resolution simulations using the same model, which tend to underestimate the amplitude of the semi permanent meander over the Bump^8^. The lower resolution simulations have topographies comparable to the topography of the SMOOTH simulation and are missing the drag induced by the lee waves as well. This wave drag might play a role in modifying the large-scale form drag and subsequently the path of the Gulf Stream over the Bump. Further diagnostics and a longer integration would be useful to understand more thoroughly the processes at stake and confirming the effects on the mean flow.

## Summary and Discussion

We observe in a realistic simulation the generation of lee waves that are in qualitative agreement with observations from satellite observations. These waves are mostly in hydrostatic and linear regime according to typical dimensionless numbers. Hence, linear theory is able to reproduce and thereby quantify the essential properties of observed lee waves, at least as far as their amplitude, wavelength of generation, and associated vertical energy flux are concerned. Furthermore, we have shown that small scale topographic features strongly impact the structure of the Gulf Stream, and that lee waves may play an active role in this topographic control. This is particularly important since these areas are most of the time omitted in global studies of lee waves.

As is well known, lee waves are a significant energy sink for the ocean circulation. Approximately 0.2 TW is converted into lee waves in the global ocean and about half of this energy is dissipated within a kilometer above the bottom^2^. Quantifying the actual amount of energy dissipated in energetic regions such as the Gulf Stream path is thus essential to understand the cascade of energy towards small scale. Further work is needed to fully quantify the impacts of lee waves generated in extreme/shallow regions, and our paper shows that high resolution numerical simulations are a valuable tool for such studies. However, these results are strongly dependent on the representation of the bathymetry in the simulations. First, small scale seamounts strongly impact the amount of lee waves radiated upward, by setting the horizontal wavelength of the waves. Second, the direction and the intensity of the mean current is modified by the smaller scales of the topography. This implies that the energy transferred through physical processes other than lee waves could be misevaluated. Third, localized overturning events (hydraulic jumps, although these cannot be well resolved in a hydrostatic simulation) can occur in the wake of seamounts (not shown in this paper), which are strongly impacted by the shape of the bathymetry. These findings can be linked with other recent studies, which also put forth the impact of small scale topography in affecting oceanic processes, such as water exchange dynamics between the Red Sea and the Gulf of Aden^23^, the evolution of internal solitary waves over the continental shelf of the South China Sea^24^, or the bi-modality of the path of the Kuroshio south of Japan^25^. These results all together therefore emphasize that an accurate representation of the bathymetry is essential to understand the ocean dynamics.

This task is all the more arduous that available high-resolution bathymetry often show suspicious patterns on the seafloor. As an example, a row of large seamounts can be seen near 31°N, 79.7°W (see gray circle in Fig. 6). In our (LEEWA) simulation, these seamounts slow down the Gulf Stream, and generate large amplitude lee waves. Moreover, small seamounts (see near 30.5°N, 78.3°W) appear suspiciously well aligned, which may result from mismatching observational data being inaccurately processed by the interpolation schemes used for bathymetry production. Such artifacts in the bathymetry would obviously result in spurious generation of lee waves and other small-scale topography-related motions. On the other hand, some real seamounts are missing in the available bathymetry datasets. For example the Stetson Mesa (30°20′N, 79°25′W), which is highlighted in ref. ^13^ as a source of internal waves, is not present in the SRTM30_PLUS dataset used in this study. Thus, as discussed in ref. ^26^, it is essential to have a better representation of the bathymetry, at a resolution down to a few hundred meters, in order to further understand the fine-scale dynamics of the ocean using high resolution numerical simulations.

## Supplementary information

Supplementary information.
