# Supplementary material for: Interaction of the Gulf Stream with small scale topography: a focus on lee waves
Source: Sci Rep. 2020 Feb 11;10:2332. doi: 10.1038/s41598-020-59297-5 (PMC7012840; doi:10.1038/s41598-020-59297-5)
Supplement: Supplementary file 1 — Supplementary information. [file 41598_2020_59297_MOESM1_ESM.pdf]

Supplementary information for "Interaction of the Gulf Stream with small scale topography: a focus on lee waves"

C. de Marez, N. J. Lahaye, and J. Gula

Introduction

The supplementary information below provide further details about methods used to describe the properties of the lee waves in the simulations.

1 Comparison between simulation output and satellite observations

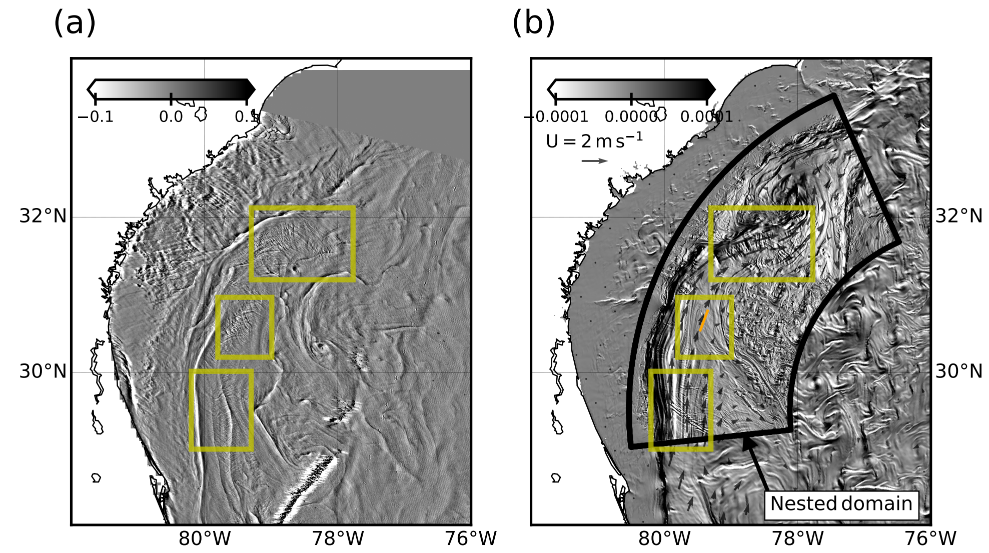

Figure 1: (a) Surface roughness [in arbitrary units] from sun glitter images measured with MODIS instrument onboard Terra satellite on 2010/04/01. (b) Snapshot of surface roughness estimate [in s^{-1}] from the LEEWA simulation. The black contour indicates the domain of the nested simulations. Yellow thick rectangles indicate areas where an important surface roughness signal likely caused by lee waves is observed in both the satellite observations and the LEEWA simulation.

2 Tools and methods used in the diagnostics of lee waves

This sections provides further details about the diagnostics performed on the simulation output.

2.1 (k, ω) spectrum calculation

Here we present how to compute the (k, ω) spectrum (dispersion diagram) of vertical velocity variance at 100 m depth ($\mathrm{w}_{100}(\mathrm{t}, \mathrm{x}, \mathrm{y})$).

Firstly, the spatial 2D Fast Fourier Transform (FFT) of $w_{100}(t, x, y)$ is calculated, which gives a real and an imaginary part $\hat{w}^r(t, k, l)$ and $\hat{w}^i(t, k, l)$ where k and l are the horizontal wave-numbers along the x- and y- axes. The temporal 1D FFT of $\hat{w}^r(t, k, l)$ and $\hat{w}^i(t, k, l)$ is then calculated. It gives two complex fields $\tilde{w}^r(\omega, k, l)$ and $\tilde{w}^i(\omega, k, l)$. To obtain the power spectral density, this quantities are recombined as follows:

$$P^w_{-100m}(\omega, k, l) = \frac{1}{4}\Big(\tilde{w}^r\,\overline{\tilde{w}^r} + \tilde{w}^i\,\overline{\tilde{w}^i}\Big) + \frac{1}{8}\Big(\mathcal{R}(\tilde{w}^i)\,\mathcal{I}(\tilde{w}^r) - \mathcal{R}(\tilde{w}^r)\,\mathcal{I}(\tilde{w}^i)\Big)$$

where $\overline{a}$ is the complex conjugate of a, and $\mathcal{R}$ and $\mathcal{I}$ are the complex and imaginary parts. Finally an azimuthal average is computed in the (k, l) space to keep only the norm of the spatial wavenumber.

2.2 Extraction of the lee waves induced pressure anomaly

This section presents how the pressure anomaly induced by the lee wave phenomena p' is extracted from the simulation outputs. The same method has been used to compute horizontal velocity anomalies u' and v'.

From a vertical along-flow section: Firstly the density is computed from the salinity S and the temperature T with the TEOS-10 equation of state. A spatial high-pass filter is then applied on the time low-passed density. The filter is applied horizontally in the direction of the section at a cutting length $\lambda_{cut} = 10\,km$. It allows to remove the density background which varies both horizontally and vertically and extract an anomaly of density ρ'. Because the filter is applied on the low-pass time-filtered field, and λ_{cut} being chosen to select the along-section small-scale variations of the topography, ρ' may be considered as the density anomaly due to lee waves. The CROCO model solving the hydrostatic primitive equations, the pressure anomaly is computed with

$$p'(z) = -\int_z^0 dz\,\rho' g. \tag{1}$$

z=0 is the surface of the ocean where the pressure anomaly is known and equal to zero.

From 3D outputs: A cubic smoothing spline algorithm is applied on the time low-passed density over each vertical level to compute the background density. The fall-off of the smoothing is chosen to keep only the small scales contribution (i.e. $O(< 10)\,km$). The anomaly of density is then obtained by removing the background. The pressure anomaly is finally obtained using hydrostatic equation (1).

2.3 Finding the typical height H and length scale L of the seamounts

We describe here how we extract typical spatial scales of the bottom seamounts from the bathymetric data, used in the estimation of the dynamical parameters ε and Fr_{lee}.

To compute the typical height scale of the bathymetry H along a section (i.e. a 1D bathymetry), we used a peak detection algorithm. Each seamount is defined by a local maximum and two minima (upstream and downstream). The difference between the maximum and the minimum is computed both upstream and downstream. Averaging the two quantities gives a mean height for each seamount. Using the same peak detection, L is defined as the distance between the lows of the bathymetry.

For a 2D bathymetry, the peak detection is performed on the de-trended topography along the x- and y- directions. The procedure follows the same line as in the 1D case: the peak/lows detection is performed along both dimensions, and the resulting 2D maps of H and L from each computation are averaged (dimension-wise) and interpolated on a regular grid.
